# Inflammatory bowel disease and extracellular matrix: when victim becomes double agent

**DOI:** 10.1007/s00011-026-02195-9

**Published:** 2026-03-17

**Authors:** Roberta Sferra, Antonella Vetuschi, Giovanni Latella, Alfredo Cappariello, Simona Pompili

**Affiliations:** 1 Department of Biotechnological and Applied Clinical Sciences, University of L’Aquila, L’Aquila, Italy; 2 Department of Life, Health and Environmental Sciences, University of L’Aquila, L’Aquila, Italy

**Keywords:** Extracellular matrix, Immune system, Inflammation, IBD, Bio-scaffold

## Abstract

**Background:**

The extracellular matrix (ECM) represents an intricate network of proteins present in all organs, with specific physical and biochemical functions. ECM is composed of two distinct but connected entities: the basement membrane, located beneath the epithelium, and the interstitial matrix, present in the mucosa and submucosa. Physiologically, ECM modulates several functions, including epithelium turnover, intercellular communications, cell adhesion, differentiation, proliferation, apoptosis, and tissue remodeling.

**Findings:**

After an injury, the epithelial barrier fails, affecting the ECM structure and functions. The normal gut structure and functions depend on ECM, which is regulated by ECM-producing cells/ECM-degrading enzymes. Intestinal injury can lead to epithelial barrier disruption and then to acute mucosal inflammation that can heal or become chronic. The ECM is directly involved in mucosal healing, while the key mechanisms leading to the chronicity of intestinal inflammation are unknown. Inflammatory cells release countless cytokines, chemokines, and growth factors, which, by interacting with specific components of the ECM, induce an overactivation of the immune system. In this context, ECM represents an important player in inflammatory diseases, including the inflammatory bowel diseases (IBD) and related complications such as intestinal fibrosis. In the last years, progressive advancements in the knowledge of IBD pathogenesis have provided crucial information for the discovery of new treatments. Nevertheless, few studies investigate the ECM’s multiple roles in the sustenance and the exacerbation of the immune reaction.

**Conclusion:**

This review aims to emphasize the dynamic aspects of the ECM, giving an overview of its direct involvement in intestinal inflammatory diseases and the related intestinal fibrosis.

## Introduction

 ECM is a dynamic, non-cellular, three-dimensional network secreted by several cells (epithelial, endothelial, fibroblasts and myofibroblasts ), having pivotal roles in cell morphogenesis, adhesion, differentiation, growth, survival, and migration [1]. ECM is a complex mixture which core matrisome includes collagens, glycoproteins and proteoglycans (PGs), assembled to form two different compartments: the *interstitial matrix (IM)*, forming a scaffolding reticulum providing support for cells in organs; and the *basement membrane* (BM), a pericellular matrix between epithelial and lamina propria, in contact with cells [2–5]. IM and BM present a different composition: IM is composed of collagen I, elastin, fibronectin, hyaluronan (HA) and PGs; while BM consists of collagen IV, laminins, heparan sulphate proteoglycans (HSPGs) and proteins secreted by epithelial, endothelial cells and myofibroblasts. BM is more compact than IM and anchored to epithelial cells *via* hemidesmosomes, making barriers modulating the passage of molecules through it [6, 7]. The ECM turnover is ruled by families of metalloproteinases (MMPs), the tissue inhibitors of metalloproteinases (TIMPs), adamalysins (ADAM-ADAMTs), meprins, proteases, as well as glycolytic enzymes (heparinase, hyaluronidases, lysyl oxidase, LOX, and other glycanases), essential for preserving ECM properties and functions [8–14]. A dysfunction of the intestinal barrier and a perturbation in the ECM structure leads to a loss of the barrier function towards the gut luminal count and then intestinal inflammation [11, 15]. Inflammatory bowel disease (IBD), including Crohn’s disease (CD) and ulcerative colitis (UC), represent an increasingly worldwide debilitating disorder, with high incidence and prevalence [15]. Its pathogenesis is influenced by various factors, including genetic predisposition, dietary habits, environmental conditions, and immune system responses, but its exact etiology remains unknown. However, changes in the intestinal homeostasis, arising from inflammation, are certainly mediated also by ECM components [16, 17]. The ECM is not an indifferent observer but an active player in IBD development. A multitude of exhaustive articles describing the causes, features and clinical outcomes of IBD are already available, thus in this review we examine the role of ECM in IBD.

## ECM in intestinal inflammation

Several pathogenic events are involved in inflammation during IBD, such as altered permeability of the epithelium/endothelium together with chemokines/cytokines activation [18]. Epithelial and ECM barrier defects facilitate the passage of bacterial and dietary antigens into the mucosa which determine the activation of immune cells [19]. This condition results in an amplified inflammatory response, a typical hallmark of IBD. The ECM of mucosa and submucosa actively participate in intestinal inflammation, and ECM remodeling is fundamental in the IBD development and progression. In fact, ECM is actively involved in intestinal inflammation both with its structural core matrisome and with its non-structural components through a complex and intricate system of interactions (Table 1). Dysregulated ECM remodeling can be caused by increased ECM production by activated myofibroblasts, as well as by changes in the expression and activation of ECM-modifying enzymes [20].

**Table 1:** ECM components fueling intestinal inflammation

ECM component	Effects	Refs
Collagen III/IV	Early increase alters type III/I ratio	[21–23]
Collagen VI	Regulates epithelial–stromal interactions	[2]
proline-glycine-proline peptide	Chemotactic for neutrophils and activates resident leukocytes	[24]
Ecm fragments	Circulating markers predictive of colitis severity	[25]
Elastin	Chemotactic via elastin-binding protein; induces Th1 differentiation and cytokine release	[26–29]
Laminin γ2 / α5	Chemotactic for neutrophils/macrophages; up-regulates TNF-α, TNFR2, MMP-9, MMP-14, uPA	[32–34]
Hyaluronan (ha)	Accumulates in inflamed colon; promotes leukocyte recruitment via CD44; increases cytokine release	[30–37]
Integrins	Cross-link between ECM and immune cells	[38–40]
Syndecan (hspg)	Decreased expression in inflamed mucosa correlates with disease severity	[42–46]
Lumican	Binds TLR-4 to sustain innate immunity; KO reduces inflammation but worsens tissue damage	[47, 48]
Mmps (metalloproteinases)	Degrade ECM, modulate chemokines, generate PGP, disrupt barrier integrity	[49– 24]
Specific mmps (e.g. mmp-1,-3,-7,-10,-13)	Alter barrier function and inflammation: MMP-13 KO preserves barrier; MMP-10 KO worsens colitis; elevated in patients	[50–56]
Adam-17	Mediates TNF-α release; increased in UC	[57, 58]
Meprin-α / meprin-β	Meprin-α KO worsens DSS colitis; Meprin-β KO protects against DSS colitis	[59, 60]
Lox (lysyl oxidase)	Up-regulated in inflamed mucosa	[61]
Heparanase	Up-regulated; activates microbiota and macrophages, promoting TNF-α release	[62, 63]
Timp-1 / timp-3	TIMP-1 increase in severe IBD; TIMP-3 KO mice more susceptible to colitis	[64–66]

### Collagens

One hallmark of the IBD is the disruption of the collagen network, generally leading to stiffness increase of the ECM and tissue. Alterations in assembly, deposition or degradation concur with pathogenesis of intestinal inflammation. During the first phase of IBD onset, the collagen type III increases, changing the ratio type III/type I [21]. High levels of Collagens III and IV are also found in experimental dextran sodium sulfate (DSS)-induced acute colitis, confirming their involvement in IBD [22, 23]. Additionally, collagen isotypes or fragments influence the resident cells’ behavior. Collagen type VI, ubiquitous across the intestinal ECM, regulates the epithelial-stromal interaction [2]. On the other hand, collagen fragments are chemotactic: the proline-glycine-proline (PGP) peptide, arising from collagen degradation, attracts neutrophils and activates resident leucocytes [24]. Interestingly, fragments deriving from collagens’ maturation as well as degradation (soluble circulating ECM) can be a suitable tool for predicting or monitoring colitis progression, being reflective for the severity and extent of disease evaluated by canonical endoscopic exploration [25].

### Elastin and laminin

Like collagen, elastin exerted chemotactic function on immune cells. Monocytes, macrophages, neutrophils and T cells express receptors called elastin-binding-protein (EBP), recognizing the X-Gly-X-X-Pro-Gly motifs. Elastin induces the differentiation of Th1 cells and the release of inflammatory cytokines [26]. Laminin (LM) represents an adhesive glycoprotein, characterized by 5 different α-chains (α1-α5), three β-chains (β1-β3), and three γ-chains (γ1-γ3). Several in vitro studies revealed that LMγ2 chain exert chemotactic effects on neutrophils. Accordingly, another study reported LMα5 chemotactic proprieties on neutrophils and macrophages. LMα5 derivates up-regulate pro-inflammatory cytokine TNF-α and its receptor TNFR2. Furthermore, peptides of LM induce the expression of MMP-9, MMP-14 and urokinase-type plasminogen activator in monocytes and macrophages [27–29].

### Glycosaminoglycans and proteoglycans

A direct correlation between ECM components and intestinal inflammation is also demonstrated by hyaluronan (HA). *Kessler et al.* reported an increased accumulation of HA in the colon samples of mice receiving DSS compared to controls, noting that HA deposition proceeds with the inflammatory response. High levels of HA are also found in human IBD biopsies, especially in blood vessels, where endothelial cells produced more HA than the control in response to tumor necrosis factor (TNF)-α exposition [30]. Other authors reported that hyaluronan, through interactions with the heavy chains of serum inter-α trypsin inhibitor, increases immune recruitment and the release of inflammatory cytokines. Other studies revealed that mice hyaluronan synthase-3 knockout (KO) (abundant in the endothelium) are protected from DSS-induced colitis by the reduction of inflammatory infiltration [20, 30–33]. These effects are mediated by the binding of HA with the CD44 on the leukocyte surface, acting as bridge between immune cells and the intestinal extracellular matrix. In IBD, inflammatory mucosa accumulates fragmented and cable-like HA, which engages CD44 on myeloid and lymphoid cells and promotes their adhesion, retention, and cytokine production within the lamina propria, thereby sustaining chronic inflammation and promoting a pro-thrombotic milieu [30, 34]. CD44 is also expressed by epithelial cells and fibroblasts, where it can modulate responses to altered matrix composition and stiffness, suggesting that CD44–hyaluronan interactions contribute more broadly to the propagation of ECM-driven inflammatory and fibrotic signals [35–37]. Additionally, both immune and stromal cells interact with ECM through integrins. Under profibrotic conditions stromal cells upregulate collagen- and fibronectin-binding integrins, which sense matrix stiffness and promote myofibroblast differentiation and excess matrix deposition. Gut-homing lymphocytes expressing α4β7 and αEβ7 (CD103) mediate adhesion to endothelial MAdCAM-1 and epithelial E-cadherin, respectively, thereby positioning effector and regulatory cells at ECM-rich interfaces [38–40]. The clinical efficacy of α4β7 blockade with vedolizumab underscores the pathophysiological relevance of these adhesion pathways [41].

Heparan sulfate proteoglycan syndecan is involved in intestinal inflammation both in DSS-induced colitis mice and in IBD patients [42–45]. A significant decrease in syndecan expression was found in the inflamed mucosa of both syndecan-1 and IL-10 KO mice [46]. A comparable reduction was noted in IBD patients correlating with the severity of the disease. Another PG involved in intestinal immune and inflammatory responses is lumican. In trinitrobenzene sulfonic acid (TNBS)-induced colitis, lumican binds toll-like receptor 4 (TLR-4) and sustains the activation of innate immunity. Lumican KO mice showed weaker inflammation but more severe tissue damage [47]. This result could be partially due to beneficial effects on wound healing exerted by lumican through interaction with the transforming growth factor-β receptor-1 (ALK-5) [48].

### ECM modifying enzymes

Exacerbated inflammatory response, characterized by progressive tissue damage, along with aberrant collagen organization, is certainly another hallmark of the IBD. The combined contribution of enzymes degrading ECM, their inhibitors as well as cytokines represent the main modulator of bowel inflammation. Several studies describe the MMPs as the main player in the ECM degradation during IBD. In the initiation of the acute inflammatory response, MMPs retrieve neutrophils and macrophages to the sites of damage influencing the activity of several chemokines and interleukins (IL-5,-6,-8) that in turn control leukocyte recruitment [49]. On the other side MMPs inactivate the chemokines action inducing an anti-inflammatory effect. Therefore, MMPs show multiple actions to chemokines, being able to activate, inactivate and antagonize them. In addition, MMPs, inducing ECM cleavage, generate several degradation products such as PGP and its acetylated form N-acetyl-(N-Ac) PGP. Deriving from collagen split, PGPs act in similar manner to chemokines recruiting neutrophils [49]. It has been demonstrated that proteolytic pathways necessary to generate PGP from collagen are activated in the intestinal tract. PGP was increased in patients with IBD [24]. Furthermore, in DSS-induced colitis in mice the inhibition of PGP leads to a significant reduction of neutrophil infiltration in intestinal wall, restoring the mucosal damage [24]. MMPs is also responsible for endothelial and epithelial barrier integrity, as MMPs activation causes the cleavage and rearrangement of cell-cell junctions and adhesion proteins resulting in migration and invasion of immune cells into the intestinal wall with an amplified inflammatory response [15]. In this context, several studies try to dissect the contribution of specific MMPs. Some authors reported high levels of MMPs expression both in experimental mouse of colitis and in IBD patients. MMP-13 KO mice showed a preserved intestinal barrier and resulted in more resistant to intestinal damage. On the contrary, MMP-10 KO mice are more prone to develop colitis. Other authors report that MMP-7 deficient mice displayed a faster renewal of the epithelium and an efficient immune response to bacteria [50, 51]. Other studies conducted on CD and UC patients assessed MMP-1,-2,-3,-14 expressions and showed a significant increase in all these MMPs, especially of MMP-1 and − 3 in IBD patients compared to healthy controls. Furthermore, studies conducted on plasma of UC patients and on children affected by CD, highlighted elevated levels of MMP-1 and MMP-3, respectively. Particularly, in children with an elevated level of MMP-3, the inflammation progression is predominant in respect to the wound healing [52, 53]. To support the MMPs involvement in the inflammatory process, other authors detected increased levels in MMP-1,-3,-7,-10 and − 12 production on the colonic epithelium of IBD patients. In biopsies from UC patients, MMP-7 and − 13 are reported to be mainly produced by vascular endothelial cells and leukocytes while MMP-8,-9 and − 10 are produced by macrophages [24, 54–56].

Some studies focused on ADAM-17, showing a direct correlation between its inhibition and a decreased release of the pro-inflammatory TNF-α [57]. However, other authors revealed a significant increase of ADAM-17 expression in colonic biopsies of UC patients compared to controls, whereas no differences were found between CD and healthy patients [58].

Several studies highlighted the involvement of meprins in the inflammatory process occurring in IBD, despite the opposite effects between the two isoforms α and β. In an experimental model of DSS-induced colitis, the intestinal damage resulted more severe in meprin-α KO compared to wild-type (WT) animals [59]. On the other hand, Meprin-β is involved in the degradation of ECM, by the release of fibronectin involved in chemiotaxis and recruitment of leukocytes, as well as in the activation of pro-inflammatory cytokines (IL-1β and IL-18). Hence, Meprin-β KO mice are less prone to develop signs of colitis after DSS administration compared to WT mice [60].

LOX represents an enzyme crucial in ECM assembly and resilience. In fact, several studies reported that LOX is involved not only in ECM remodeling in physiological conditions, but also in the development of inflammation, fibrosis and tumor microenvironments [61, 62]. However, the data about LOX action in IBD is still limited. In a mouse model of TNBS-induced colitis, *Rivera et al.* reported that LOX levels were significantly increased in inflamed mucosa compared to healthy tissue. The result supports the hypothesis of an association between this enzyme and the IBD pathogenesis [62]. In 2011, *Lerner et al.* revealed the heparanase contribution in the bowel inflammation. The study highlighted an increase in the expression of this enzyme both in human UC and CD and in experimental models of DSS-induced colitis, compared to the healthy tissues. The authors reported that heparanase stimulated the intestinal flora and induced macrophages activation that in turn induced the activation of pro-inflammatory cytokines (i.e. TNF-alpha) sustaining the development of the colitis [63].

An important role in IBD pathogenesis is also exerted by MMPs inhibitors, despite TIMPs involvement remains still controversial. Some authors showed a positive correlation between high serum levels of TIMP-1 and severity of human IBD patients’ plasma. On the contrary, other studies revealed no difference in TIMP-1 expression in IBD tissue samples that showed an increase in levels of MMP-3 [64]. Other authors revealed high levels of TIMP-1, but not of TIMP-2 in myofibroblasts isolated from CD lesions compared to healthy bowel [65]. Another study noted TIMP-3 KO resulted more susceptible to experimental colitis compared to controls [66].

## Interactions between ECM-producing cells and cytokines in IBD

The alteration of the epithelial barrier leads to the activation of immune cells and the release of cytokines that not only trigger the inflammatory response but also affect the behavior of the ECM-depositing cells. Upon exposition to these cytokines, many cells type over the body, such as fibroblasts, subepithelial myofibroblasts, smooth muscle cells, epithelial, endothelial cell, stellate cells and pericytes, switch their phenotype and become activated ECM-producing myofibroblasts, representing the principal source of extracellular matrix (ECM) in IBD-associated fibrosis [67, 68]. Experimental work in intestinal fibrosis models and human IBD tissues has further suggested that a proportion of ECM-producing cells may arise from epithelial or endothelial compartments via epithelial- and endothelial-to-mesenchymal transition (EMT and EndoMT) [69, 70]. However, this contribution remains debated and, to date, is supported mainly by in vitro systems, organoid and animal models, and marker- or transcriptomics-based analyses rather than definitive lineage-tracing evidence in patients, so EMT/EndoMT should be still considered potential but not yet firmly established sources of intestinal myofibroblasts in human IBD. In this context, Fibroblast Activation Protein positive (FAP⁺) fibroblasts were recently identified as the key subtype with the highest level of ECM production in fibrotic intestines. The increased infiltration of FAP⁺ fibroblasts in fibrotic sites has been revealed by single-cell analyses in resected Crohn’s ileum and murine colitis, where macrophage-derived IL-1β/TGF-β trigger pathologic matrix deposition and stricture formation by TWIST1⁺/FAP⁺ fibroblasts [71]. Pharmacologic or genetic TWIST1 inhibition reduces fibrosis, nominating this axis for anti-fibrotic strategies in CD [71]. Similarly, in fibro-stenotic Crohn’s disease spatial transcriptomics showed a tight colocalization of FAP⁺ fibroblasts with CD150⁺ inflammatory monocytes in the submucosa of inflamed/stenotic ileum, but not in healthy or ulcerative colitis tissue [72]. CD150⁺ monocytes secrete high levels of IL-1α/β, TNF and TGF-β1, which in turn drive TWIST1 expression and activation of FAP⁺ fibroblasts, creating a feed-forward inflammatory–profibrotic niche [72].

Several cytokines are pleiotropic factors physiologically involved in the homeostasis of all tissues and are also responsible for the severe systemic side effects of IBD (Table 2).

**Table 2:** Cytokines involved in intestinal inflammation

Member	Effects	Refs
IL-1β	Recruits granulocytes; activates innate lymphoid cells	[73, 74]
IL-18	Elevated in CD; blockade ameliorates colitis; promotes IFN-γ, IL-1β, TNF-α	[75]
IL-33	Acute phase: worsens via IL-1RL1; in chronic phase: Th1 suppression and Th2 induction	[76]
IFN-γ	Drives TNF-α, IL-1β, IL-6 production; IFN-γ KO reduces inflammation	[77, 78]
Type I IFNs	Receptor deficiency worsens colitis	[79]
IL-6	Via sIL-6R STAT3 activation, prevents apoptosis, boosts pro-inflammatory cytokines; blockade increases T-cell apoptosis, and reduces cytokine production	[80, 81]
IL-22	Activates IECs via STAT3; promotes mucosal healing, goblet-cell recovery, mucus production	[82, 83]
TNF-α / TNF–ECM complexes	Pro-inflammatory via TNFR1/2 activation of NF-κB, angiogenesis, Paneth cell necroptosis, MMP induction, immune-cell activation, IEC damage; binds fibronectin/laminin to store/regulate active TNF; enhances CD4⁺ T-cell adhesion via β1 integrin	[84–89]
TGF-β	Latently stored in ECM (via LTBPs); activated by proteases, integrins, thrombospondin driving fibroblast to myofibroblast differentiation, ECM deposition (collagen, fibronectin, PGs); induces Treg differentiation producing anti-inflammatory IL-10/TGF-β; reduced signaling (e.g. Smad7 increase) induces colitis	[90–102]

### IL-1 superfamily

IL-1 signaling is highly regulated by the binding with its receptor antagonist (IL-R1a). In fact, studies conducted on IBD patients highlight high levels of IL-R1a both in plasma and in tissues. These results support the hypothesis that this antagonist takes part in hosting defense mechanism against the inflammation. Furthermore, increased levels of IL-1 are found in IBD, resulting in an activation of IL-1 converting enzyme (ICE) and in the release of mature IL-1 β in the colon [73]. Accordingly, other authors reported that IL-1β exert a key role in the onset of colonic inflammation, recruiting granulocytes and activating innate lymphoid cells (ILCs) [74].

IL-18 involvement in IBD is reported both in experimental and human studies. In fact, an inhibition of IL-18 in mice colitis models resulted in an amelioration of the inflammatory progression. Moreover, high levels of these cytokines are found in CD patients compared to controls [75]. This increase correlated to the activation of other pro-inflammatory cytokines (interferon: IFN-γ, IL-1β and TNF-α).

IL-33 plays a phase-dependent action in experimental models of DSS-induced colitis. In acute inflammation IL-33 leads to a worsening of colitis signs through the activation of IL-1RL1 signaling.

On the contrary, it exerts a beneficial effect in the chronic phase of the disease suppressing Th1 response and inducing Th2 immune reaction. Increased levels of IL-33 production were found in UC patients compared to healthy bowel [76].

### Interferons

IFN-γ certainly represents the best characterized isoforms of IFN in experimental model of colitis. A significant increase in this cytokine is found in DSS-induced colitis in mice compared to control. Accordingly, IFN-γ knockout animals showed a significant reduction in inflammation induced by DSS [77]. These data confirm that INF-γ plays an important role in IBD pathogenesis. Another study reported that INF-γ, released by Th1 cells, stimulated the production of TNF-α and IL-1B and IL-6 [78]. The involvement of INF family in gut inflammation, is also demonstrated by *Katakura et al.* in an experimental model of colitis with mice lacking type I IFN receptor. The authors report that deficient animals showed marked signs of inflammation respect to wild-type group [79].

### IL-6 superfamily

IL-6 is physiologically involved in the differentiation of intestinal epithelial cells and in wound healing. IL-6 increased in experimental models of colitis and human IBD exerting a pro-inflammatory action. This function is mediated by the activation of several cells, such as antigen presenting cells and T cells. IL-6 binds to the soluble form of its receptor (sIL-6R), forming a complex (IL-6-sIL-6R) activating signal transducer activator of transcription (STAT)-3 pathway that prevents apoptosis and induces the production of other pro-inflammatory cytokines [80]. Accordingly, studies conducted inhibiting the IL-6 signaling in a mouse model of colitis revealed the increase of T cells apoptosis, correlated with a significant decrease of pro-inflammatory cytokines (IFN, TNF, IL-1β) in inflamed tissues. These data support the use of drugs (i.e. tocilizumab) able to block IL-6 signaling, as potential pharmacological therapy in CD patients [81].

Other authors, using a Th2-mediated model of colitis, demonstrated that supplementary administration of IL-22 exerts beneficial effect on local bowel inflammation [82]. On the contrary, in double KO for both T- and B-cells, no features of amelioration were found [83]. IL-22 can induce the activation of intestinal epithelial cells (IECs) via STAT3 signaling leading to a recovery of the inflamed mucosa in experimental model of DSS-induced colitis [84]. IL-22/STAT3 signaling facilitates the re-formation of goblet cells and the release of mucus, necessary for the correct epithelial barrier [85].

## Interactions between ECM components and cytokines in IBD

The alteration of the intestinal barrier leads to the passage into the mucosa of intraluminal dietary and microbial antigens that can induce an anomalous and excessive immune response responsible for the inflammatory process [86]. In response to barrier alterations, several types of cells release pro-inflammatory (i.e. IL1β, IL6, TNF) and anti-inflammatory (i.e. IL10, TGF-β) cytokines. In the intestinal inflammation ECM components interact with several cytokines, enhancing cell-ECM interactions. However, this aspect is still poorly investigated.

### Tumor necrosis factor

TNF-α, a key effector of the immune response, is a transmembrane protein cleaved in soluble form by metalloproteinase TNF-converting enzyme (ADAM-17). Increased production of both the two ADAM-17 isoforms by CD14 + macrophages, adipocytes, fibroblasts, and T cells, was found in IBD patients. TNF-α acts as pro-inflammatory cytokine in colitis through the interaction with its receptors (TNFR1 and 2) inducing the activation of the transcription factor nuclear factor-kB (NF-kB). This event increased angiogenesis, necroptosis of Paneth cells, increased production of MMPs, induced activation of macrophages and T cells and damage of IECs. Furthermore, TNF-α cooperates with two important members of ECM, laminin, and fibronectin. The strict binding between TNF-α and these molecules leads to a complex stored in the ECM where regulates the viability of the TNF active form [87]. Particularly, TNF-α/fibronectin interaction increases the adhesion of activated lymphocytes CD41, mediated by β1 integrin [88]. Similarly, TNF-α/laminin complex results in a significant increase of cell adhesive properties regulated by β1 integrin. This event results in the activation of CD4 + T cells [89]. Other authors revealed that high levels of the α isoform of TNF in the inflamed tissue induced the secretion of other pro-inflammatory cytokines IL-1 and IL-6, of adhesion molecules, immune cells and MMPs, generating a positive feedback loop that supply constantly the inflammatory process [90–92].

### Transforming growth factor-β

TGF-β, is a cytokine involved in several functions, including embryogenesis, tissue homeostasis, cell proliferation, differentiation, immune regulation, and then inflammation, fibrogenesis, and carcinogenesis. Furthermore, TGF-β stimulates the production of several ECM molecules, such as fibronectin, collagen, and PGs. It is therefore directly involved in matrix deposition and the development of intestinal fibrosis [93]. TGF-β is secreted by cells in a latent form binding with latent binding proteins (LTBPs). Since LTBPs are present into the ECM, this latter entraps and stores the latent TGF-β and regulates its activation and mobilization [94]. Among latent TGF-β activators there are ECM components, such as proteases, thrombospondin-1 and integrins [95, 96]. Tissue injury leads to ECM loss integrity with consequent release of activators of latent TGF- β, Activated TGF-β can induce the differentiation of fibroblasts in myofibroblasts enhancing their proliferation and resistance to apoptosis. Activated myofibroblasts produce ECM molecules as collagen type I, fibronectin, and TIMP-1 [97]. However, the role of TGF-β is complex, especially in the context of the inflammation and fibrosis [98, 99]. In fact, although TGF-β released by macrophages exerts a pro-fibrotic effect, in the inflammatory process it is produced by T cells and plays a beneficial anti-inflammatory action. Particularly, TGF-β induces the differentiation of CD4 + T_reg_ cells that in turn produce IL-10 and TGF-β to attenuate inflammation [100]. Accordingly, experimental models of mice lacking T_reg_ cells releasing TGF-β spontaneously developed intestinal inflammation [101]. Studies on IBD patients showed that reduced activity of TGF-β, due to high expression of Smad7, leads to the development of colonic inflammation [102].

Another central profibrotic axis during IBD progression involving TGF-β comprises YAP/TAZ pathway. Beyond canonical SMAD2/3 signaling, TGF-β increases nuclear YAP/TAZ, which normally operates as a mechanosensitive transcriptional system that integrates biochemical cues with mechanical features of the intestinal microenvironment [103]. Once activated, YAP/TAZ regulates fibroblast proliferation, survival, ECM production, finally driving tissue repair in inflammatory settings [104]. In IBD, these functions become pathological. Persistent YAP/TAZ activation in intestinal fibroblasts promotes excessive deposition of collagen and other ECM components, increasing tissue stiffness and fueling a feed-forward loop of mechano-transduction and inflammation. In Crohn’s disease, YAP/TAZ-high fibroblast populations are enriched within fibrotic strictures, where they directly contribute to ECM expansion and luminal narrowing [105]. Accordingly, experimental colitis studies show that inhibiting YAP/TAZ reduces fibrosis and suppresses myofibroblast-related gene expression [106].

## ECM biomimetic as a therapeutic tool for IBD

Biomimetics of ECM scaffolding is a novel approach to treating the IBD as demonstrated by several studies in animal models of IBD [107, 108]. Biomimetic ECM scaffolding has several advantages over other treatments for IBD, such as a better mimetic of the natural structure and function of the intestinal ECM, which provides mechanical support, biochemical signals, and cell adhesion sites for the intestinal epithelium, and on the other hand reduces inflammation, and prevent fibrosis [109, 110]. Moreover, EMC mimics allow an enhancement of the healing effects, since they can be combined with therapeutic agents, such as growth factors, anti-inflammatory drugs, or stem cells. They also can be delivered to the affected area with minimally invasive methods (i.e. by injection, implantation, or endoscopy) [111, 112]. Finally, they avoid the side effects and drawbacks of traditional treatments using autologous or exogenous tissues, such as infections, immunogenicity, rejection or shortage of donors [6]. Biomimetic ECM scaffolding is a new method for intervention in intestinal bowel diseases having the purpose to recreate the structure and function of the ECM. The scaffolding can be obtained by using decellularized tissues or fabricated by means of biomimetic materials, e.g. collagen, hyaluronic acid, or synthetic polymers [113]. The advantages of scaffolds are promoting tissue regeneration, relieving inflammation, and preventing fibrosis. Biomimetic ECM scaffolding can be provided to the target sites via injection, implantation, or endoscopy, and other therapeutic agents, like growth factors, anti-inflammatory drugs, or stem cells, can be used in conjunction. It has been shown by multiple studies that bionic ECM scaffolding may be a very promising intervention in animal models of IBD, achieving the higher re-epithelialization of the damaged epithelium, the lower the inflammation and the more restoration of the epithelial barrier function. Altogether the studies performed nowadays indicate that biomimetic ECM scaffolding is a particularly promising tool to restore intestinal homeostasis of IBD patients.

## Conclusions

The intestinal epithelium lies on BM, a specialized matrix consists of a dense filamentous carpet with numerous fenestrations that promote water, ions, and nutrients transport. Furthermore, forming a connection with the overlying epithelial cells, BM acts as an important physical and immunological barrier against food and gut microbiome antigens, as well as against to pathogenic organisms [20]. The mucosal and submucosal layers consist of a complex network of fibroblasts, smooth muscles cells, blood and lymphatic vessels and nerves that maintain the tissue architecture. The mucosal and submucosal ECM is constituted by diagonal collagen fibrils and fibronectin responsible for elasticity required during peristalsis and regulation of cellular behavior migration and proliferation. For a long time, the ECM was considered only an inactive structural component of tissue and organs. Nowadays, it is well known that this aspect is only one of the multiple facets of the ECM.

The intestinal ECM therefore behaves as an exemplary “double-edged sword” [114, 115]. During acute injury, tightly regulated deposition of a provisional matrix and its subsequent degradation are indispensable to restore epithelial continuity, re-establish barrier function and support immune cell and endothelial trafficking. In this phase, ECM remodelling is largely adaptive and pro-resolution. However, when inflammatory cues are repetitive or chronic, the same reparative programs drive persistent activation of mesenchymal cells, progressive accumulation and cross-linking of collagen and other matrix components and stiffening of the bowel wall. This transition from physiological wound healing to maladaptive fibrosis underlies stricture formation in Crohn’s disease and increased wall stiffness with motility disturbances in ulcerative colitis [116]. This double behavior falls not only on ECM structural network but also on immune cells homeostasis [117]. In fact, experimental and human data support a partial uncoupling of inflammation and ECM remodelling, in which profibrogenic pathways can remain active even when mucosal inflammation is controlled. In physiological condition, the ECM maintains a specific amount and composition through a balanced action of enzymes degrading ECM components and their inhibitors. In this view, the ECM shows its “light side”, representing a dynamic structure crucial in vital processes such as intercellular communications, wound healing and tissue remodeling. However, there is another side of the coin: after an injury, ECM equilibrium is perturbated leading to the excessive activation of the immune response. Leukocytes start to release pro-inflammatory cytokines; ECM degradation becomes uncontrolled and in turn ECM releases other inflammatory factors. These events trigger a vicious circle constantly nourished that are responsible for the maintenance and progression of the chronic inflammatory process as occurs in IBD, as well as the related complications such as the possible development of fibrosis and cancer. Although this “dark side” of the ECM is emerging, this aspect is less investigated. In the last few years, a lot of progress has been made in the control of intestinal inflammation in patients with IBD, however no effective and well-tolerated drugs are available to prevent the development of intestinal fibrosis or to treat it once it has occurred, fibrosis which is responsible for organ failure. This conceptual framework from “light-to dark side” of ECM is critical to interpret anti-inflammatory treatment responses that achieve endoscopic or even histological remission but fail to normalise bowel wall mechanics.

A further challenge for clinical management in IBD is that ECM signatures and their trajectories differ not only between CD and UC, but also across their respective phenotypic spectra. In clinical practice, Crohn’s disease behaviour is commonly classified according to the Montreal system into non-stricturing, non-penetrating (B1), stricturing (B2) and penetrating (B3) phenotypes. Histological and biomarker data indicate that ECM remodelling is not restricted to “complicated” B2/B3 disease. In a detailed transmural analysis of resected ileal Crohn’s disease, Tavares de Sousa et al. showed that the overall degree of transmural fibrosis was remarkably similar across B1, B2 and B3 phenotypes, whereas stricturing disease was characterised by more pronounced muscularis propria thickening and myenteric plexus alterations rather than simply greater collagen deposition [118]. Complementary serological studies using neo-epitope assays of collagen formation and degradation demonstrated distinct collagen turnover signatures across Montreal classes, with specific ECM biomarker profiles associated with stricturing and penetrating behaviour and predictive of future disease progression [119, 120]. These observations suggest that many B1 patients already harbour substantial, and at least initially reversible, ECM remodelling despite the absence of fixed strictures or fistulas. Whether a patient remains clinically in B1 or progresses to B2/B3 is likely to reflect whether this remodelling resolves together with inflammation or crosses critical biomechanical thresholds resulting in irreversible matrix accumulation, cross-linking and smooth muscle hypertrophy. Framing ECM biology within the Montreal classification therefore helps to link molecular fibrogenic pathways to the heterogeneous clinical trajectories of Crohn’s disease.

On the other hand, UC is classically described as a mucosa-confined disease, but several studies have highlighted that fibrosis of the submucosa and deeper layers is common and clinically relevant. In a systematic histopathological mapping of 89 colectomy specimens from patients with long-standing UC, Gordon et al. detected submucosal fibrosis in 100% of colonic segments (706 cross-sections) [121]. Fibrosis burden and thickening of the muscularis mucosae closely correlated with markers of chronic mucosal injury, but not with active inflammation, and were associated with increased wall stiffness. These structural changes are therefore plausible contributors to motility disturbances, urgency and incontinence even in the absence of overt endoscopic inflammation.

The advent of intestinal ultrasound (IUS) and elastography has enabled in-vivo characterization of these structural alterations in UC. Transabdominal ultrasound with shear-wave elastography shows that measures of wall stiffness and shear-wave velocity correlate with disease activity but can remain elevated in a subset of patients in clinical or endoscopic remission, consistent with persistent fibrosis [122, 123]. Recent IUS studies have also emphasized the importance of assessing individual wall layers, with submucosal thickness and hyper-echogenicity emerging as particularly responsive to treatment and reflective of underlying structural damage [123]. Clinically, this helps to explain why some UC patients in apparent remission continue to experience diarrhoea, urgency or abdominal discomfort and may eventually require surgery, while colectomy specimens in such cases often reveal pronounced submucosal fibrosis with only minimal residual inflammation. Incorporating ECM-related parameters—such as submucosal thickness and stiffness—into disease assessment may therefore refine our definition of remission in UC and provide a framework for the development of antifibrotic therapeutic strategies.

**Fig. 1:**
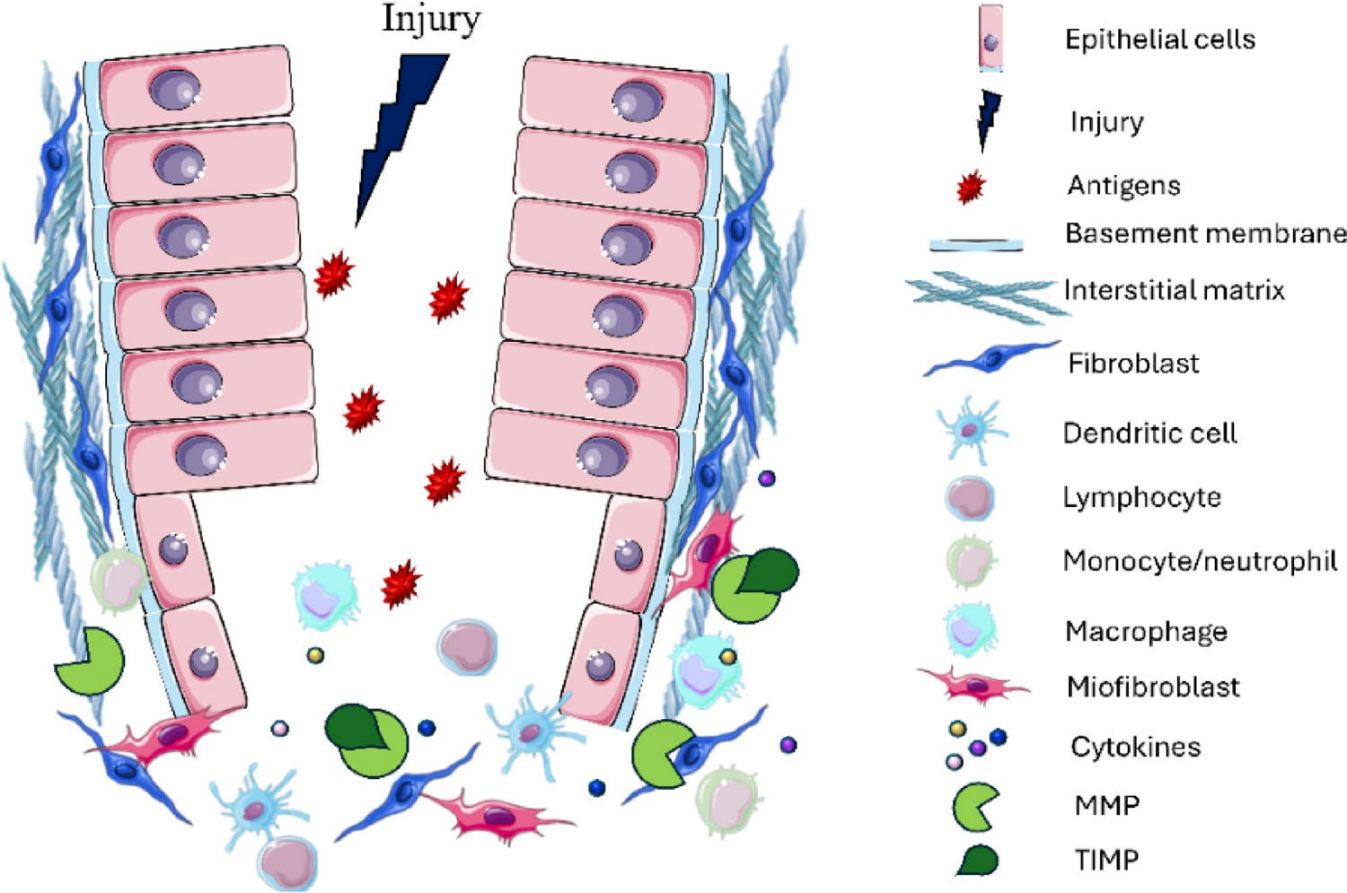
ECM in intestinal inflammation. Schematic representation of intestinal mucosa and the ECM involvement in intestinal inflammation. After an injury, the epithelial barrier fails, allowing free access to antigens. Immune cells including dendritic cells, recognize the antigens and release pro-inflammatory cytokines, recruiting lymphocytes, monocytes/neutrophils, and macrophages. Concomitantly, in ECM basement membrane and interstitial matrix the equilibrium between enzymes degrading (MMPs) and their inhibitors (TIMPs) is unbalanced. MMPs levels increase, ECM components are degraded and other inflammatory cytokines, stored in ECM, are released. Therefore, the immune response is overactivated and the inflammatory process is constantly nourished. Finally, if the inflammation protracts over time, fibroblasts present in ECM switch their phenotype in activated myofibroblasts, leading to the development to the chronic inflammation related fibrosis

Currently, up to 75% of CD patients are still forced to undergo surgery mainly for intestinal stenosis, but also for cancer; for this reason, IBDs represent a global health problem on which many efforts are converging to strike an effective pharmacological therapeutic strategy [124, 125]. Nowadays, the drugs available for the IBD treatment, such as salicylates, immunosuppressor, biological agents relieve the inflammation without ameliorating the ECM-related events like pro-fibrotic process and the risk of cancer development. An alternative approach under investigation in experimental models of IBD is the use of anti-inflammatory agents with antifibrotic molecules, such as hepatocyte growth factor (HFG), bone morphogenetic protein-7 (BMP-7) and peroxisome proliferator-activated receptors (PPARs) modulator [126, 127]. Many future expectations are placed in the biomimetic ECM as a therapeutic tool for IBD. The purpose of biomimetic ECM scaffolding is to recreate the structure and function of the ECM and to restore intestinal homeostasis and functions in IBD patients. Therefore, in this review we have tried to highlight the wide range of ECM roles in chronic intestinal inflammation (Fig. 1). The indirect effects that ECM-producing cells exert in the release of pro-inflammatory molecules, as well as the direct interactions between ECM components on the one hand with the immune system and on the other with the epithelial cells and overall, with the barrier functions of the mucosa are an important element in the IBD clinical outcomes. To focus the attention on the ECM components and to operate to maintain ECM integrity could be the outsider to limit the progression of the inflammation and to reduce or eliminate its complications, opening the possible avenue for the identification of new therapeutic targets.

## Data Availability

No datasets were generated or analysed during the current study.
